# Association between 25-Hydroxyvitamin D and Metabolic Syndrome in Older Adults: The Health, Aging and Body Composition Study

**DOI:** 10.1155/2021/6671823

**Published:** 2021-03-13

**Authors:** Subhashish Agarwal, Janet A. Tooze, Douglas C. Bauer, Jane A. Cauley, Tamara B. Harris, Annemarie Koster, Catherine R. Womack, Stephen B. Kritchevsky, Denise K. Houston

**Affiliations:** ^1^Department of Cardiology, Medical College of Wisconsin, Milwaukee, WI, USA; ^2^Division of Public Health Sciences and Sticht Center on Aging, Wake Forest School of Medicine, Winston-Salem, NC, USA; ^3^General Internal Medicine, University of California San Francisco, San Francisco, CA, USA; ^4^Department of Epidemiology, University of Pittsburgh, Pittsburgh, PA, USA; ^5^Laboratory of Epidemiology, Demography, and Biometry, National Institute on Aging, Bethesda, MD, USA; ^6^Department of Social Medicine, University of Maastricht, Maastricht, Netherlands; ^7^Department of Preventive Medicine, University of Tennessee Health Science Center, Memphis, TN, USA; ^8^Sticht Center on Aging, Wake Forest School of Medicine, Winston-Salem, NC, USA; ^9^For the Health ABC Study, Sticht Center on Aging, Wake Forest School of Medicine, Winston-Salem, NC, USA

## Abstract

**Objective:**

Low 25-hydroxyvitamin D (25[OH]D) levels and metabolic syndrome (MetS) are prevalent among older adults; however, longitudinal studies examining 25(OH)D status and MetS are lacking. We explore the association of 25(OH)D levels with prevalent and incident MetS in white and black older adults. *Research Design and Methods*. A total of 1620 white and 1016 black participants aged 70–79 years from the Health ABC cohort with measured 25(OH)D levels and data on MetS and covariates of interest were examined. The association between 25(OH)D levels and prevalent MetS at baseline and incident MetS at 6-year follow-up was examined in whites and blacks separately using logistic regression adjusting for demographics, lifestyle factors, and renal function.

**Results:**

At baseline, 635 (39%) white and 363 (36%) black participants had prevalent MetS. In whites, low 25(OH)D levels were associated with prevalent MetS (adjusted OR (95% CI), 1.85 (1.47, 2.34)) and 1.96 (1.46, 2.63) for 25(OH)D of 20–<30 and <20 vs. ≥30 ng/ml, respectively). The association was attenuated after adjustment for BMI but remained significant. No association was found between 25(OH)D levels and prevalent MetS in blacks. Among those without MetS at baseline (765 whites, 427 blacks), 150 (20%) whites and 87 (20%) blacks had developed MetS at 6-year follow-up. However, 25(OH)D levels were not associated with incident MetS in whites or blacks.

**Conclusion:**

In older adults, low 25(OH)D levels were associated with increased odds of prevalent MetS in whites but not in blacks. No association was observed between 25(OH)D levels and incident MetS in either whites or blacks.

## 1. Introduction

Vitamin D in some studies has been shown to improve bone health, prevent osteoporosis, bone fractures, and boost immunity and perhaps lower the risk for diabetes [1, 2]. However, low 25-hydroxyvitamin D [25(OH)D] levels are common worldwide, with higher prevalence in individuals residing in northern latitudes [3]. In the US, the prevalence of low 25(OH)D levels (below 30 ng/ml) increased from 55% to 77% between 1988 and 2004 [4]. Older adults (due to lower 7-dehydrocholesterol levels in the skin and decreased outdoor activity), women (due to lower outdoor activity), and individuals with dark skin (due to increased melanin) have a higher prevalence of low 25(OH)D levels [4–8]. Forty to one hundred percent of USA and European community-dwelling older adults have been reported to have deficient 25(OH)D levels [9, 10].

In several studies involving young to middle age populations, low 25(OH)D levels have been associated with metabolic syndrome (MetS) [11–13] and its components such as hypertension [14], obesity [15], and insulin resistance [16, 17]. Higher dietary intake of vitamin D was also inversely related to MetS and its components in younger adults [18, 19]. Although older adults have a high prevalence of low 25(OH)D levels [4, 7] and MetS [20, 21], the association between the two has not been thoroughly investigated. Cross-sectional evidence regarding the relationship between 25(OH)D and MetS is inconsistent with some studies showing an association between low 25(OH)D levels and MetS in older adults [22–24] and others showing no association [25]. Moreover, previous studies are limited by their inclusion of low numbers of nonwhite older adults. The current study explores both cross-sectional and longitudinal associations between 25(OH)D levels and metabolic syndrome in the Health, Aging and Body Composition Study, a community-based study of well-functioning white and black older adults.

## 2. Research Design and Methods

The Health, Aging and Body Composition (Health ABC) study is a longitudinal, prospective cohort study investigating the associations among body composition, weight-related health conditions, and incident functional limitations in older adults. The Health ABC study cohort consists of 3075 well-functioning black and white men and women aged 70–79 at baseline (1997-1998). White participants were recruited from a random sample of Medicare beneficiaries in Pittsburgh, Pennsylvania, Memphis, Tennessee, and the surrounding area. Black participants were recruited from all age-eligible residents of the areas in and surrounding Pittsburgh and Memphis. Eligibility included reporting no difficulties performing activities of daily living (ADL), walking a quarter of a mile, or climbing 10 steps without resting. In addition, all participants were free of a terminal diagnosis and had no intention to move from the area for at least 3 years. All study participants provided written informed consent prior to participation.

Data collection for the present analysis occurred during the periods 1997–1999 and 2002-2003. Participants with measured 25(OH)D at the 12-month follow-up visit served as the baseline population for this analysis (*n* = 2793). Among those with measured 25(OH)D, 2760 participants had complete data for classification of metabolic syndrome at baseline. After exclusion of participants with missing covariates (*n* = 124), 1620 white and 1016 black participants had complete data for analysis. After excluding participants with prevalent metabolic syndrome at baseline (*n* = 635 whites, *n* = 363 blacks) and those missing data at 6-year follow-up (*n* = 446; *n* = 160 deceased, *n* = 206 with a home, phone, or proxy interview, *n* = 33 lost to follow-up or withdrew, and *n* = 47 missing MetS components), 765 white and 427 black participants had complete data for classification of incident metabolic syndrome at follow-up.

### 2.1. Classification of Metabolic Syndrome

The National Cholesterol Education Program/Adult Treatment Panel III (NCEP ATP III) [26, 27] definition was used to classify participants having MetS in the Health ABC cohort at the baseline and 6-year follow-up visit. Three of the five components are required for diagnosis: (1) waist circumference ≥102 cm (men) and ≥88 cm (women), (2) hypertension ≥130 mm Hg systolic or ≥85 mm Hg diastolic or antihypertensive medications, (3) fasting blood glucose ≥100 mg/dl or treatment for impaired fasting glucose, (4) triglycerides ≥150 mg/dl or specific treatment, and (5) HDL-C ≤40 mg/dl in men and ≤50 mg/dl in women. Waist circumference was measured using a flexible measuring tape on bare skin at the level of maximal circumference, midway between the lower ribs and anterior superior iliac spine at the level of the umbilicus by trained technicians. Seated systolic and diastolic blood pressures were measured by a manual mercury sphygmomanometer using a standardized protocol. Fasting plasma glucose levels were measured by an automated glucose oxidase reaction (YSI 2300 glucose analyzer, Yellow Springs Instruments, Yellow Springs, Ohio). Triglyceride and high-density lipoprotein (HDL) cholesterol concentrations were measured by a colorimetric technique (Johnson and Johnson Vitros 950 analyzer, Rochester, New York).

### 2.2. Assessment of 25(OH)D and PTH

Serum 25(OH)D was measured at the 12-month follow-up visit (1998-99) in blood samples collected in the morning after a 12-hour fast, centrifuged, and stored at −80°C. Serum 25(OH)D was measured using a 2-step radioimmunoassay (25-hydroxyvitamin D 125I RIA Kit, DiaSorin, Stillwater, Minnesota, USA). The interassay coefficient of variation for serum 25(OH)D was 6.78%. Intact parathyroid hormone (iPTH) was measured in EDTA plasma with a two-site immunoradiometric assay kit (N-tact PTHSP, DiaSorin, Stillwater, Minnesota, USA). The interassay coefficient of variation for plasma iPTH was 8.6%.

### 2.3. Covariates

Sociodemographic factors (age, gender, race, study site, and education), smoking history, alcohol consumption, and weekly physical activity from walking and exercise (minutes/week) were determined by interview-administered questionnaires. Dietary intake was measured at the 12-month follow-up visit with a 108-item semiquantitative food frequency questionnaire (FFQ). This FFQ was designed specifically for the Health ABC study by Block Dietary Data Systems (Berkeley, CA) and administered by trained dietary interviewers. Vitamin D-containing supplements were defined as multivitamins and supplements containing 3 or fewer ingredients, one of which was vitamin D. Height and weight were obtained using a Harpenden stadiometer (Holtain, Wales, UK) and a standard balance beam, respectively. Body mass index (BMI) was calculated as weight divided by height squared (kg/m^2^). Due to fluctuations of 25(OH)D levels with season, a season variable was created (winter, December–February; spring, March–May; summer, June–August; and fall, September–November). 25(OH)D is metabolized in the kidneys by the enzyme 25-hydroxyvitamin D-1*α*-hydroxylase (CYP27B1) to its active form, 1,25-dihydroxyvitamin D; hence, we adjusted for renal function [28]. Serum creatinine was assayed by a colorimetric technique on a Johnson and Johnson Vitros 950 analyzer. Prevalent diabetes was defined by the self-reported history of adult onset of diabetes, fasting glucose of 126 mg/dl or greater, or use of insulin or oral glucose-lowering medications.

### 2.4. Statistical Analysis

Participant characteristics were compared by MetS status stratified by race using the *t* test for continuous variables and chi-square for categorical variables. The association between 25(OH)D levels (<20 ng/ml, 20–<30 ng/ml, and ≥30 ng/ml) and prevalent MetS and each of the MetS components at baseline and incident MetS at 6-year follow-up was examined using multivariable logistic regression adjusting for age, gender, site, season, education, smoking, alcohol use, physical activity, creatinine, and total energy intake (model 1) plus BMI (model 2). Tests for a linear trend were conducted by entering the median 25(OH)D value of each category (<20 ng/ml, 20–<30 ng/ml, and ≥30 ng/ml). Two-way interactions between gender and 25(OH)D levels and between race and 25(OH)D levels were tested. Significant interactions were found for race and 25(OH)D levels (*P*=0.04); thus, all analyses are stratified by race. Because diabetes has been associated with low 25(OH)D levels, a separate sensitivity analysis was performed after exclusion of those with prevalent diabetes at baseline. Finally, in a separate analysis, we further adjusted for parathyroid hormone (PTH) to determine if the association between 25(OH)D and MetS is independent of PTH. All statistical analyses were performed using JMP version 8 (SAS Institute Inc., Cary, North Carolina) with statistical significance at *P* < 0.05 using two-sided tests.

## 3. Results

**Figure 1:**
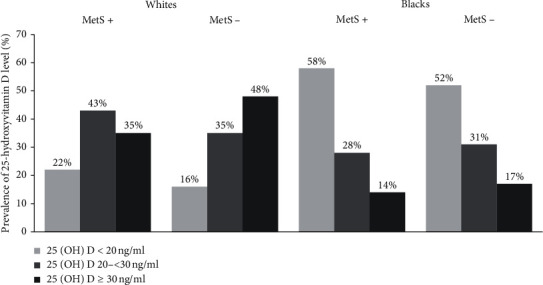
The distribution of 25-hydroxyvitamin D levels in white and black participants classified by the presence or absence of prevalent metabolic syndrome at baseline in the Health ABC cohort (*n* = 2636). 25(OH)D, 25-hydroxyvitamin D; MetS, metabolic syndrome.

A total of 1620 white and 1016 black participants with 25(OH)D levels at 12-month follow-up were examined with a mean (SD) age of 73.7 (2.9) and 73.4 (2.9) years at baseline, respectively. At baseline, 635 (39%) white and 363 (36%) black participants had MetS. The mean (SD) 25(OH)D level in whites was 29.1 (10.3) ng/ml with a range from 5.6 ng/ml to 104.5 ng/ml. The mean (SD) 25(OH)D level in blacks was 20.9 (10.3) ng/ml with a range from 5.0 ng/ml to 186.9 ng/ml. The distribution of 25(OH)D levels by race and presence or absence of metabolic syndrome is presented in Figure 1. In whites, the mean serum 25(OH)D concentration in those with MetS was significantly lower compared to participants without MetS (mean serum 25(OH)D concentration, 27.4 vs. 30.2 ng/ml, *P* < 0.0001). In blacks, the mean serum 25(OH)D concentration in participants with and without metabolic syndrome was not significantly different (Table 1). Whites with MetS were less likely to be physically active and to report alcohol consumption and were more likely to have lower levels of education and a higher creatinine and BMI compared to those without MetS. Blacks with MetS were more likely to be women, have a higher BMI and be current smokers, and were less likely to report alcohol consumption but were more likely to be current smokers compared to those without MetS (Table 1). Dietary calcium and vitamin D intake and vitamin D supplement use were similar between those with and without MetS. As expected, the mean value of each component of MetS was higher among those with MetS except for systolic blood pressure in blacks.

### 3.1. 25(OH)D and Prevalent MetS

In whites, the odds of prevalent MetS was significantly higher among those with 25(OH)D levels of 20–<30 and <20 ng/ml compared to those with 25(OH)D levels ≥30 ng/ml after adjusting for age, gender, site, season, education, smoking status, alcohol use, physical activity, serum creatinine, and total energy intake (*P* for trend = 0.0001; Table 2). The association was attenuated but remained statistically significant after further adjustment for BMI. When models were further adjusted for PTH, 25(OH)D levels of 20–<30 and <20 ng/ml remained significantly associated with increased odds of prevalent MetS compared to 25(OH)D ≥30 ng/ml in whites (OR (95% CI), 1.59 (1.24, 2.04) and 1.48 (1.07, 2.05), respectively). There was no association between 25(OH)D and prevalent MetS in blacks (Table 2). In separate sensitivity analyses after excluding individuals with diabetes (*n* = 167 whites, and *n* = 207 blacks), 1453 whites and 809 blacks were evaluated, and the patterns of the association between 25(OH)D and prevalent MetS were qualitatively similar to the overall association observed between 25(OH)D and prevalent MetS in each race when both diabetic and nondiabetic participants were included (data not shown).

Additionally, we examined whether 25(OH)D was associated with the presence of individual MetS components at baseline in both white and black participants (Table 3). Among the MetS components, significant associations were observed between low 25(OH)D levels and abdominal adiposity, hypertension, hyperglycemia, elevated triglycerides, and low HDL cholesterol among whites. However, low 25(OH) D levels were only associated with abdominal adiposity in blacks (Table 3).

### 3.2. 25(OH)D and Incident MetS

Of the 1192 people free of MetS at baseline who had MetS assessed at the 6-year follow-up visit, there were 237 incident cases of MetS (whites, *n* = 150/765, 20%; blacks, *n* = 87/427, 20%). Incident MetS was not associated with 25(OH)D levels after adjusting for age, gender, site, season, education, smoking status, alcohol use, physical activity, serum creatinine, and total energy intake (model 1) or BMI (model 2) in either white or black participants (Table 4). Similarly, no association between 25(OH)D and incident MetS was observed after excluding participants with prevalent diabetes at baseline (results not shown).

## 4. Discussion

In this cohort of healthy, well-functioning older adults, low 25(OH)D levels were associated with greater prevalence of MetS independent of demographics, lifestyle factors, and renal function in whites but not in blacks. Furthermore, no association was observed between low 25(OH)D levels and incident MetS in either whites or blacks. Associations between 25(OH)D levels and both prevalent and incident MetS were similar in participants without prevalent diabetes at baseline.

Several cross-sectional studies [11, 13, 22, 24, 29, 30] have shown an increased prevalence of MetS in participants with low 25(OH)D levels. Oosterwerff et al. [24] examined white men and women aged 65–88 years from the Longitudinal Aging Study Amsterdam (LASA) and found those with 25(OH)D <20 ng/ml had a 29% higher odds of prevalent MetS compared to those with 25(OH)D >20 ng/mL. However, no adjustment was made for BMI. Similarly, Reis et al. [11] examined men and women aged 20 y and older using data from the 2003-2004 NHANES and found those with 25(OH)D ≥35 ng/ml had a 74% lower odds of prevalent MetS independent of confounders including calcium intake and PTH, and the association was unaffected by age or gender.

In contrast, a few studies [25, 31] show no association between 25(OH)D levels and MetS. In the Rancho Bernardo study [25], no association between 25(OH)D levels and MetS was demonstrated in a cross-sectional analysis of older men and women. The authors concluded that this discrepancy could be the result of higher 25(OH)D levels (mean 25(OH)D: 43.6 ng/ml in men, 40.7 ng/ml in women) in a population residing in a sunny and temperate year-round climate, reducing the variability observed in 25(OH)D levels.

In the present study, associations between 25(OH)D levels and prevalent MetS were observed in cross-sectional analyses. However, this association was significant in whites but not in blacks. The mean 25(OH)D levels for blacks and whites were 21.0 ng/ml and 29.1 ng/ml, respectively. However, even with lower 25(OH)D levels in blacks, no association with prevalent MetS was seen in contrast to the NHANES data where the association between 25(OH)D and prevalent MetS was similar in Caucasians, African Americans, and Mexican Americans [30]. However, this discrepancy could be due to inclusion of both young and middle age populations in NHANES data compared to our study population of older adults. MetS is a constellation of interrelated risk factors whose underlying pathophysiology is associated with insulin resistance. To further understand the racial differences observed between 25(OH)D and MetS, we examined the associations between 25(OH)D and MetS components in whites and blacks. Elevated waist circumference, hypertension, hyperglycemia, low HDL, and elevated triglycerides were significantly associated with low 25(OH)D levels in whites; whereas in blacks, only elevated waist circumference was significantly associated with low 25(OH)D levels in the Health ABC cohort. Additionally, whites had significantly higher triglycerides and lower HDL compared to blacks, perhaps resulting in an association between low 25(OH)D levels and prevalent MetS in whites in the Health ABC cohort.

A recent study [32] in Australia found an increased odds of 41% for development of incident MetS in middle-aged whites with 25(OH)D <18 ng/ml compared to participants in the highest quintile of 25(OH)D ≥34 ng/ml. However, this association was attenuated and was no longer significant after further adjustment for BMI and insulin resistance. In contrast, in our analysis, no association between 25(OH)D levels and incident MetS was seen in either white or black older adults indicating that perhaps 25(OH)D may not be involved in insulin resistance pathways and development of new MetS. Moreover, aging leads to arteriosclerosis, essential hypertension, and MetS; thus, this physiology in older adults perhaps acts as a confounder as MetS is present irrespective of the 25(OH)D level.

The strength of the present study is the large cohort of healthy, well-functioning older adults with approximately equal representation of women and blacks which leads to a broader generalizability of our findings. However, various limitations need to be acknowledged. The observational nature of our study does not allow us to evaluate a causal association between 25(OH)D and MetS. Although experimental evidence suggests a role for 25(OH)D in glucose homeostasis with 25(OH)D <20 ng/ml associated with insulin resistance [16] and obesity [15], this association does not in itself translate into increased risk of metabolic syndrome as it is possible that higher BMIs often observed in individuals with MetS leads to lower 25(OH)D levels. It is interesting to note that although animal data in basic and preclinical studies provide a useful backdrop to hypotheses arguing for nonskeletal effects of vitamin D, human studies in terms of several meta‐analyses, as well as recent RCTs, have not been designed to permit any definitive conclusions [33, 34].

A potential limitation is that 25(OH)D levels were measured at the 12-month follow-up visit and not at baseline when prevalent MetS was assessed. Another significant limitation is the lack of 25(OH)D assay standardization. In fact, vitamin D research data are plagued by variation in the quality of serum total 25(OH)D assay methods which has compromised and continues to compromise the ability to distinguish among the different guidelines currently in use [34].

Moreover, only a single measurement of 25(OH)D was obtained which limits inference regarding long-term 25(OH)D exposure. Thus, the lack of association seen between 25(OH)D and incident MetS in this analysis may be hampered by the absence of interim 25(OH)D levels. Components of the MetS were only measured in the clinic at baseline and the 6-year follow-up visit. We only had data on MetS in 77% of whites and 65% of blacks at follow-up after excluding prevalent MetS at baseline and participants who did not come for a clinic visit. The status of incident MetS in participants who did not attend the clinic visit or were lost to follow-up is unknown, perhaps leading to loss of power and deduction abilities.

In conclusion, our results suggest that low 25(OH)D levels may increase the odds for prevalent metabolic syndrome in older adults, particularly in whites. This association appears independent of numerous potentially confounding factors including total energy intake, BMI, and renal function. However, no association was observed between low 25(OH)D levels and incident metabolic syndrome in older white or black men and women. Additional prospective studies are needed to determine whether low 25(OH)D levels are associated with metabolic syndrome. Furthermore, randomized clinical trials are necessary to determine whether treating patients with low 25(OH)D levels with supplemental vitamin D reduces the risk of MetS. If a causal link between 25(OH)D and MetS is established in older adults, then low 25(OH)D levels may become a practical target for prevention and treatment due to its high prevalence, ease of detection, and availability of inexpensive and effective treatment.

## Figures and Tables

**Table 1:** Baseline characteristics of the Health ABC participants with and without the presence of metabolic syndrome at baseline by race (1997-98).

Variables	Whites (*n* = 1620)		*P* value	Blacks (*n* = 1016)		*P* value
	MetS present	MetS absent		MetS present	MetS absent	
Demographics						
Total participants	635 (39%)	985 (61%)		363 (36%)	653 (64%)	
Age (y)	73.6 (2.8)	73.8 (2.9)	0.09	73.2 (2.7)	73.5 (2.9)	0.07
Male (%)	319 (50%)	533 (54%)	0.13	123 (34%)	316 (48%)	≤0.0001

Site						
Memphis	327 (51%)	495 (50%)	0.63	161 (44%)	309 (47%)	0.36
Pittsburgh	308 (49%)	490 (50%)		202 (56%)	344 (53%)	

Education						
Less than high school	83 (13%)	107 (11%)	0.01	141 (39%)	280 (43%)	0.40
High school graduate	233 (37%)	308 (31%)		119 (33%)	192 (29%)	
More than high school	319 (50%)	570 (58%)		103 (28%)	181 (28%)	

Alcohol intake						
None	292 (46%)	365 (37%)	0.0003	251 (69%)	391 (60%)	0.02
Low (<1 drink/week)	140 (22%)	205 (21%)		69 (19%)	142 (22%)	
Moderate (1–7 drinks/week)	157 (25%)	311 (32%)		31 (9%)	84 (13%)	
High (>7 drinks/week)	46 (7%)	104 (11%)		12 (3%)	36 (5%)	

Smoking						
Never	275 (43%)	427 (43%)	0.82	168 (46%)	297 (45%)	0.02
Former	326 (51%)	498 (51%)		156 (43%)	246 (38%)	
Current	34 (5%)	60 (6%)		39 (11%)	110 (17%)	

Physical activity (minutes walking/week)						
0 minutes	239 (38%)	304 (31%)	0.02	188 (52%)	312 (48%)	0.24
1–149 minutes	202 (32%)	334 (34%)		106 (29%)	188 (29%)	
≥150 minutes	194 (30%)	347 (35%)		69 (19%)	153 (23%)	

BMI category						
Normal weight (<25 kg/m^2^)	134 (21%)	483 (49%)	<0.0001	32 (9%)	238 (36%)	<0.0001
Overweight (25–<30 kg/m^2^)	309 (49%)	412 (42%)		130 (36%)	262 (40%)	
Obese (≥30 kg/m^2^)	192 (30%)	90 (9%)		201 (55%)	153 (23%)	

Other measures						
Serum creatinine (mg/dl)	1.06 (0.56)	0.99 (0.20)	0.005	1.11 (0.41)	1.10 (0.38)	0.83
Total energy intake (Kcal/day)	1819 (679)	1822 (650)	0.92	1885 (826)	2030 (970)	0.01
Total calcium intake (mg/day)	782.6 (360.3)	816.8 (405.8)	0.08	762.0 (427.5)	758.6 (407.9)	0.90
Vitamin D intake (IU/day)	212.3 (131.3)	222.3 (140.7)	0.15	194.4 (146.5)	193.7 (142.0)	0.94
Vitamin D-containing supplement use	305 (48%)	477 (49%)	0.73	97 (27%)	174 (27%)	0.97
Serum 25(OH)D (ng/ml)	27.4 (9.7)	30.2 (10.6)	<0.0001	20.4 (12.7)	21.2 (8.7)	0.28
Parathyroid hormone (pg/ml)	37.3 (19.3)	33.8 (15.9)	0.0001	47.3 (36.2)	43.8 (35.1)	0.13

MetS criteria						
Waist circumference (cm)	104.2 (11.5)	95.2 (11.4)	<0.0001	107.6 (11.4)	96.6 (13.8)	<0.0001
Systolic blood pressure (mmHg)	137.9 (20.2)	130.9 (19.3)	<0.0001	139.6 (21.4)	138.2 (22.5)	0.35
Diastolic blood pressure (mmHg)	70.6 (11.0)	69.4 (11.0)	0.03	72.1 (11.8)	74.1 (12.6)	0.02
Fasting blood glucose (mg/dl)	111.8 (34.3)	93.1 (17.0)	<0.0001	131.7 (53.5)	95.6 (19.6)	<0.0001
Triglycerides (mg/dl)	211.2 (101.9)	113.9 (45.6)	<0.0001	157.9 (100.8)	97.1 (35.1)	<0.0001
High-density lipoprotein (mg/dl)	43.5 (12.5)	57.5 (16.2)	<0.0001	48.7 (14.4)	62.0 (17.2)	<0.0001

*P* value was obtained by the *t* test for continuous variables and chi-square for categorical variables stratified by race. Continuous variables are reported as mean (standard deviation) and categorical variables reported as frequency (percentages). BMI, body mass index; MetS, metabolic syndrome; IU, international units; 25(OH)D, serum 25-hydroxyvitamin D.

**Table 2:** Association between 25-hydroxyvitamin D levels and prevalent MetS at baseline in the Health ABC cohort by race.

	Whites				Blacks			
	25(OH)D < 20 ng/ml	25(OH)D, 20–<30 ng/ml	25(OH)D ≥ 30 ng/ml	*P* for trend	25(OH)D < 20 ng/ml	25(OH)D, 20–<30 ng/ml	25(OH)D ≥ 30 ng/ml	*P* for trend

Prevalence of MetS	138 (46%)	274 (44%)	223 (32%)	<0.0001	210 (38%)	102 (33%)	51 (32%)	0.10
Model 1	1.96 (1.46, 2.63)	1.85 (1.47, 2.34)	1.00	0.0001	1.22 (0.83, 1.80)	0.96 (0.63, 1.46)	1.00	0.17
Model 2	1.58 (1.16, 2.17)	1.63 (1.27, 2.08)	1.00	0.0003	0.95 (0.63, 1.43)	0.82 (0.52, 1.28)	1.00	0.95

Data represent number (percentage) and odds ratios (OR) with 95% confidence intervals (CI). Model 1, adjusted for age, gender, site, season, education, smoking status, alcohol use, physical activity, serum creatinine, and total energy intake. Model 2, adjusted for all variables in model 1 plus body mass index. 25(OH)D, 25-hydroxyvitamin D.

**Table 3:** Association between 25-hydroxyvitamin D levels and MetS components at baseline in the Health ABC cohort by race.

Race	Whites			Blacks		
25(OH)D levels	25(OH)D < 20 ng/ml	25(OH)D, 20–<30 ng/ml	25(OH)D ≥ 30 ng/ml	25(OH)D < 20 ng/ml	25(OH)D, 20–<30 ng/ml	25(OH)D ≥ 30 ng/ml
Elevated waist circumference						
Model 1	2.02 (1.48–2.76)	1.42 (1.12–1.79)	1.00	1.77 (1.16–2.69)	1.17 (0.75–1.84)	1.00
Model 2	1.56 (1.07–2.27)	1.13 (0.85–1.49)	1.00	1.31 (0.79–2.19)	0.94 (0.54–1.61)	1.00

Hypertension						
Model 1	1.41 (1.01–1.97)	1.34 (1.04–1.72)	1.00	0.89 (0.53–1.50)	0.93 (0.53–1.62)	1.00
Model 2	1.35 (0.96–1.89)	1.29 (1.00–1.67)	1.00	0.84 (0.50–1.42)	0.91 (0.52–1.60)	1.00

Impaired fasting glucose						
Model 1	2.08 (1.44–3.00)	1.76 (1.30–2.37)	1.00	1.15 (0.77–1.72)	0.79 (0.51–1.23)	1.00
Model 2	1.85 (1.27–2.68)	1.61 (1.19–2.19)	1.00	0.98 (0.65–1.49)	0.72 (0.46–1.13)	1.00

Elevated triglycerides						
Model 1	1.57 (1.18–2.11)	1.43 (1.14–1.80)	1.00	1.01 (0.63–1.62)	1.29 (0.78–2.13)	1.00
Model 2	1.40 (1.04–1.88)	1.32 (1.04–1.67)	1.00	0.89 (0.55–1.45)	1.21 (0.73–2.01)	1.00

Low HDL cholesterol						
Model 1	1.69 (1.24–2.30)	1.84 (1.44–2.34)	1.00	1.38 (0.87–2.20)	1.40 (0.86–2.29)	1.00
Model 2	1.50 (1.10–2.06)	1.70 (1.33–2.18)	1.00	1.26 (0.79–2.02)	1.33 (0.81–2.19)	1.00

Data represent odds ratios (OR) with 95% confidence intervals (CI). Model 1, adjusted for age, gender, site, season, education, smoking status, alcohol use, physical activity, serum creatinine, and total energy intake. Model 2, adjusted for all variables in model 1 plus body mass index.

**Table 4:** Association between 25-hydroxyvitamin D levels and incident MetS at 6-year follow-up in the Health ABC cohort by race.

	Whites				Blacks			
	25(OH)D < 20 ng/ml	25(OH)D, 20–<30 ng/ml	25(OH)D ≥ 30 ng/ml	*P* for trend	25(OH)D < 20 ng/ml	25(OH)D, 20–<30 ng/ml	25(OH)D ≥ 30 ng/ml	*P* for trend

Incidence of MetS	25 (22%)	56 (20%)	69 (18%)	0.33	46 (22%)	27 (19%)	14 (20%)	0.62
Model 1	1.18 (0.68, 2.04)	1.17 (0.78, 1.76)	1.00	0.44	1.16 (0.57, 2.34)	0.92 (0.44, 1.94)	1.00	0.57
Model 2	0.98 (0.55, 1.74)	1.02 (0.67, 1.56)	1.00	0.99	1.06 (0.51, 2.21)	0.96 (0.45, 2.06)	1.00	0.82

Data represent number (percentage) and odds ratios (OR) with 95% confidence intervals (CI). Model 1, adjusted for age, gender, site, season, education, smoking status, alcohol use, physical activity, serum creatinine, and total energy intake. Model 2: adjusted for all variables in model 1 plus body mass index. 25(OH)D, 25-hydroxyvitamin D.

## Data Availability

The data used to support the findings of this study are provided by the Health ABC study (https://healthabc.nia.nih.gov/).
